# Caregivers’ perceptions of the oral-health-related quality of life of children with special needs in Johannesburg, South Africa

**DOI:** 10.4102/hsag.v24i0.1056

**Published:** 2019-09-23

**Authors:** Cathrine Nqcobo, Tshakane Ralephenya, Yolanda M. Kolisa, Temitope Esan, Veerasamy Yengopal

**Affiliations:** 1 Department of Paediatric and Restorative Dentistry, University of the Witwatersrand, Parktown, South Africa; 2 Department of Community Dentistry, University of the Witwatersrand, Parktown, South Africa; 3 Department of Restorative Dentistry, Faculty of Dentistry, Obafemi Awolowo University, Ile-Ife, Nigeria

**Keywords:** Oral-health-related quality of life, Dental caries status, Caregiver perception, Children with special needs, Oral health

## Abstract

**Background:**

The prevalence of dental caries and its effect on the oral-health-related quality of life (OHRQoL) of children with special needs (CSNs) have not been established in South Africa.

**Aim:**

The study aimed to assess how caregivers of CSNs who attended Down Syndrome Association outreach sites in Johannesburg, South Africa, perceived the contribution of OHRQoL to the quality of life of these children.

**Setting:**

The study was conducted at Down Syndrome Association (DSA) outreach sites in Johannesburg. These sites cater for children with several types of disabilities including cerebral palsy, hydrocephalus, autism, epilepsy and developmental delays. The association schedules and facilitates support group meetings for the caregivers of children with Down syndrome and other disabilities. These meetings are held at the outreach sites that are located at different district hospitals and community health centres in Johannesburg and are co-facilitated by the association’s outreach coordinator together with a team of physiotherapists, occupational therapists and speech therapists.

**Methods:**

This cross-sectional study was composed of a convenient sample of 150 caregiver and child pairs from five outreach sites during January – June 2015. The short-form Parent-Caregiver Perception Questionnaire (P-CPQ) was used. The caries status of the children was assessed using the decayed, missing and filled teeth (dmft/DMFT) indices (whereby dmft or DMFT stands for decayed missing filled teeth in primary dentition [dmft] and in permanent dentition [DMFT]) based on World Health Organization guidelines.

**Results:**

The mean age of the caregivers was 39.52 years (standard deviation [SD] 9.26) and 8.72 years (SD 6.07) for the children. The mean P-CPQ score was 12.88 (SD 12.14). All the caregivers stated that dental caries had a negative impact on the OHRQoL of the CSNs. However, 60% of caregivers stated that an oral condition had no impact on the child’s overall well-being. The majority (56.7%) of the caregivers rated their children’s overall oral health status as average and only 12% reported the oral health status to be poor. There was a high prevalence of untreated caries among the CSNs regardless of the type of disability.

**Conclusion:**

All the caregivers stated that dental caries had a negative impact on the OHRQoL of the CSNs. However, they appeared to have contradictory perceptions of the oral health needs or status of their children.

## Introduction

Over a billion people (15% of the world’s population) are estimated to live with some form of disability and this percentage is on the increase (WHO 2014). In South Africa, about 7.5% of the population is regarded as having disabilities with the lowest prevalence (5%) reported in Gauteng province (STATSSA 2014). Studies show that the prevalence of disabilities is higher among women (8.3%) than men (6.5%). About 2.1 million children in South Africa (11.2% of the total child population) are categorised as children with disabilities that is children with special needs (CSNs). Of the total child population with special needs, 28% is in the 0–4-year-old group and 10% is in the 5–9-year-old group (STATSSA 2014).

Children with special needs are the neglected segment of the population in terms of access to services like education and health. However, there is no up-to-date data in South Africa about their general health status and the use of health services (UNICEF 2012). Current data show that attendance at early childhood development centres or schools among children aged 5–6 years with disabilities is lower than for those without disabilities (STATSSA 2014) and children with disabilities have poor access to oral health care services.

Limitations such as motor, sensory and intellectual disabilities lead to CSNs having difficulties in maintaining oral health and communicating their oral health needs. As a result, they depend on their caregivers for general care including oral hygiene (Oredugba & Akindayomi 2008). Studies have reported that caregivers of CSNs also face an increased burden because of the demands of taking care of their children. In some instances, the huge burden related to the general health concerns of these children often results in the neglect of their oral health as this is not regarded as a priority (Lewis et al. 2015).

Oral-health-related quality of life (OHRQoL) is defined as:

The impact of oral diseases and disorders on aspects of everyday life that a patient or person values, that are of sufficient magnitude, in terms of frequency, severity or duration to affect their experience and perception of their life overall. (Locker & Allen 2007:409)

Evidence has shown that untreated caries is higher in CSNs (Nqcobo et al. 2012). Given that untreated dental caries can lead to difficulty with eating, speech, pain, sleep disturbances and missed days at school (Sheiham, Conway & Chestnutt 2015), it may contribute to poorer OHRQoL outcomes (Cushing, Sheiham & Maizels 1986).

The caregivers’ perceptions of children’s oral health status and OHRQoL influence their oral health-care-seeking behaviour and motivates them to access oral health services (Pradhan 2013; Vann et al. 2010). Caregiver perception of their children’s oral health is often used as a proxy measure (Baghdadi 2014; Pani et al. 2013) of the child’s OHRQoL. A range of factors such as caregivers’ gender, monthly family income, mother’s education, family structure and increased caregiver stress have an impact on the caregiver’s perception of the OHRQoL (Baghdadi 2014; Cushing et al. 1986; Pani et al. 2013)

The OHRQoL outcomes of CSNs in Johannesburg have not been established in the literature. The aim of this study was therefore to assess the OHRQoL outcomes because of dental caries rate among CSNs.

## Research methods and design

### Study setting

The study was conducted at Down Syndrome Association (DSA) outreach sites in Johannesburg. These sites cater for children with several types of disabilities including cerebral palsy, hydrocephalus, autism, epilepsy and developmental delays. The association schedules and facilitates support group meetings for the caregivers of children with Down syndrome and other disabilities. These meetings are held at the outreach sites that are located at different district hospitals and community health centres in Johannesburg, and are co-facilitated by the association’s outreach coordinator together with a team of physiotherapists, occupational therapists and speech therapists.

### Study design, study population and sample

This cross-sectional study consisted of a convenient sample of caregiver and child pairs. Caregivers were defined as ‘all the parents, legal guardian or relatives’ who attend to the needs of a dependent child with special needs (Family Caregiver Alliance 2012). The participants were recruited between January and May 2015 from the DSA support groups, and from a special needs school in Johannesburg.

The DSA in Johannesburg has outreach sites which cater for children with several types of disabilities including cerebral palsy, hydrocephalus, autism, epilepsy and developmental delays. The association schedules and facilitates support group meetings for the caregivers of children with Down syndrome and other disabilities. These meetings are held at the outreach sites that are located at different district hospitals and community health centres in Johannesburg, and are co-facilitated by the association’s outreach coordinator together with a team of physiotherapists, occupational therapists and speech therapists. During the group meetings, the caregivers have facilitated group discussions and peer group education sessions wherein several topics are discussed, for example, caring for a child with Down syndrome and other disabilities and how to stimulate children with disabilities, developmental milestones, diet, oral health care, speech development and support, occupational health as well as social grant support issues and challenges, and so on. All the caregivers attending the support group meetings were invited to participate in the study and their children were also enrolled in the study.

At the end of a 4-month period of data collection, only 37% of the sample was collected as a result, a decision was made to purposively recruit the caregivers from a special needs school in Johannesburg which was visited by the department outreach team at the time of data collection. The researchers attended the school open day where parents were invited to participate in the study. The parents gave consent to have their children participate in the study and have an oral examination during school days. The children’s clinical assessment form and the parent or caregivers questionnaires were matched and identifiers were kept confidential so that participants were not identifiable to persons not involved in the study.’ According to the STATA 12 statistical sample size calculator, the sample size was calculated to be 150 caregiver–child pairs including the 20 attrition effect and the variables used were alpha of 0.05 and 80% power.

### Data collection and study instrument

This study used the validated short-form Parent-Caregiver Perception Questionnaire (P-CPQ) (Thomson et al. 2013) which aims to assess parental perceptions of their children’s OHRQoL. The questionnaire consisted of 16 closed-ended questions grouped into four subscales or domains: oral symptoms (OS), functional limitations (FLs), emotional well-being (EW) and social well-being (SWB) domains. The questionnaires were administered by a trained interviewer who explained and gave clarifications to the caregivers. Training was essential to ensure the uniformity of the questioning by reducing interviewer error. The questionnaire was piloted at a site that was not included in the study. The caregivers also answered two questions that were related to the child’s OHRQoL global rating score. This score summarises how the caregivers perceive the children’s OHRQoL using two questions and is often used to test for construct validity. The first question asks the caregivers to rate their children’s overall oral health status (health of the teeth, lips, jaws and mouth) using a five-point Likert scale and the response options are from ‘excellent’ to ‘poor’. The second question focuses on how the overall well-being of the child is affected by the oral health status using a Likert scale ranging from ‘not at all’ to ‘very much’ (Jokovic, Locker & Guyatt 2005). When the global rating score correlates well with the perceived quality of life score, it indicates that the OHRQoL perception score is valid.

### Clinical examination of the children

Dental caries was measured using the decayed, missing and filled teeth (dmft or DMFT) indices whereby dmft or DMFT stands for decayed missing filled teeth in primary dentition (dmft) and in permanent dentition (DMFT). Two calibrated examiners conducted the clinical examination. Inter- and intra-examiner reliability scores were assessed using the Cohen Kappa scores which were found to be 0.9 for diagnosis of dental caries. The children were examined on site under natural light, using a mouth mirror, in a seated position according to the modified World Health Organization guidelines (WHO 2013). The assessment form was used to record dmft or DMFT data and to collect information on the socio-demographic status of the child and the caregiver.

### Data analysis

Data from the questionnaires were captured into Microsoft Excel and analysed using the Statistical Package for Social Sciences (SPSS) version 16. The independent variables were the demographic variables (caregiver age and gender), socio-economic condition (household income, caregiver’s education level and employment status) and clinical status (prevalence of untreated dental caries and dmft or DMFT scores). The dependent variable was the OHRQoL outcomes as determined by the P-CPQ total overall and domain scores. The scores were calculated from a five-point Likert scale and all the scores in each domain were added separately to give a domain score. The sum of the four domain scores made up the total P-CPQ score for each child. The scores ranged from 0 to 64 and the lower scores represented a high OHRQoL.

Descriptive statistics was used to describe the demographic profile of the caregiver–child pairs using means, standard deviations, frequency and ranges of the total and domain scores. *T*-test, Mann–Whitney and one-way analysis of variance (ANOVA) tests were carried out to assess the group differences between means and the Games–Howell post-hoc tests were also used to identify group differences. The statistical significance level was set at *p* < 0.05 and estimates were reported at the 95% confidence interval.

### Ethical considerations

Permission to undertake the study was granted by the University of Witwatersrand Ethics Committee (Ethics Clearance Certificate number M140438), the Department of Education and the Downs Syndrome Association Johannesburg branch. Informed consent was obtained from all individual caregivers included in the study. The caregiver of each participant was given an information sheet and a written consent form for obtaining their permission to allow their children to participate in the study which they had to sign.

## Results

### Demographics

The study consisted of 150 caregiver and child pairs and the profile of the study participants is shown in Table 1. The mean age of the caregivers was 39.5 years (standard deviation [SD] 9.26) and 94.7% of the caregivers were women. Of these female caregivers, 87% were mothers to the children. The mean age of the children was 8.72 years (SD 6.07) and men were the predominant gender (59.30%). The majority of the caregivers (48%) had secondary education and 52.70% of them were employed. Analysis of source of income indicated that the majority of the caregivers (64.0%) earned a salary, while 27.30% received a monthly care dependency grant (a grant which a primary care giver applies for on behalf of a child who needs permanent care because of disability). Only 8% of the caregivers received a child support grant, that is, a grant that can be applied for by needy caregivers to help support the care of a child irrespective of health status (Table 1).

**TABLE 1:** Frequency and percentage of caregivers by socio-demographic characteristics.

Variable	Frequency (*n*)	Percentage (%)
**Caregiver gender**	**150**	**100**
Female	142	94.7
Male	8	5.3
**Child gender**	**150**	**100**
Female	61	40.70
Male	89	59.30
**Level of education**	**150**	**100**
No schooling	2	1.30
Primary (Grades 1–7)	14	9.30
High school (Grades 8–12)	72	48.00
College	34	22.70
University	25	16.70
Others	3	2.00
**Employment status**	**150**	**100**
Not employed	60	40.00
Employed	79	52.70
Self employed	5	3.30
Other	6	4.00
**Source of income**	**150**	**100**
Salary	96	64.00
Care dependency grant	41	27.30
Pension grant	1	0.70
Child grant	12	8.00

**FIGURE 1:**
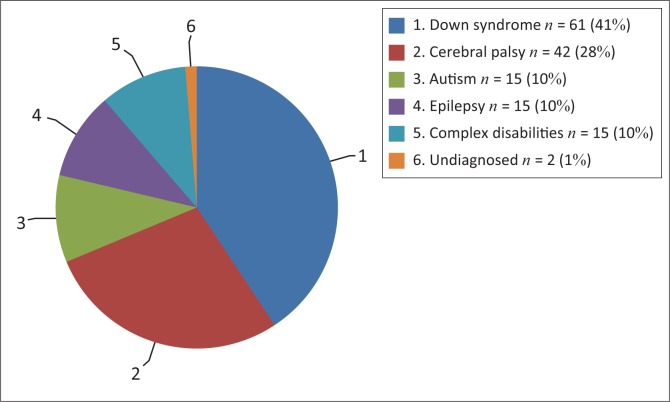
Number and percentage of children by type of disability.

Figure 1 provides information about the distribution of the child participants according to type of disability. The majority of the children had Down syndrome (41%), while 10% of the children had disabilities which are grouped as complex disabilities. These include conditions like myotonic dystrophy (*n* = 5), severe physical disability (*n* = 6) and syndromes such as Noonan syndrome (*n* = 2) and Cornelia de Lange syndrome (*n* = 2).

### Dental caries status

**FIGURE 2:**
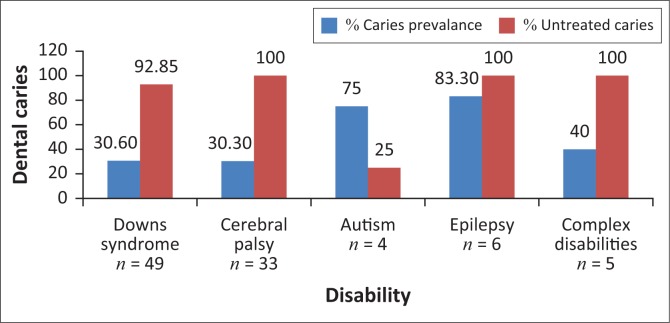
Dental caries prevalence and untreated caries in primary dentition by disability.

The overall caries prevalence was 42% and the dmft score was found to be 1.45 (SD 2.58). Figure 2 provides information on the caries prevalence and untreated caries in the primary dentition of the participants in each of the disabilities. The highest caries prevalence was found in the epilepsy (83.3%) and the autism groups (75%) compared to Down syndrome and cerebral palsy. The untreated caries remained high (93% – 100%) across all the disabilities regardless of caries prevalence and disability.

### Oral health relate quality of life

The data for the OHRQoL are reported based on the overall as well as domain scores of the P-CPQ as indicated in Table 2. The majority of the caregivers (91%) indicated that oral conditions had a negative impact on the OHRQoL (P-CPQ > 0). The overall mean P-CPQ score was relatively lower (12.88 SD 12.14) as it ranged from 0 to 44. High scores were found in all the domains except the SWB domain (2 SD 3.0). There was no significant difference in the mean P-CPQ scores among the different disabilities.

**TABLE 2:** Mean, standard deviations, ranges of domains and total parent-caregiver perception (Parent-Caregiver Perception Questionnaire) scores.

Domains and overall P-CPQ scores	Mean	SD	Expected range	Observed range
Oral symptoms	4.62	3.91	0–16	0–16
Functional limitations	4.38	4.06	0–16	0–14
Emotional well-being	3.95	4.02	0–16	0–16
Social well-being	2.23	3.00	0–16	0–11
**P-CPQ**	**12.88**	**12.14**	**0–64**	**0–44**

P-CPQ, Parent-Caregiver Perception Questionnaire; SD, standard deviation.

Tables 3 and 4 show that there were significant differences in the domain scores of OS, functional limitation domain as well as the overall OHRQoL between those individuals with dental caries and those without caries in primary dentition. Those with dental caries expressed a higher negative impact on their quality of life, whereas those with caries in the permanent dentition only expressed a greater negative impact on the OS domain compared to those with a caries-free dentition.

**TABLE 3:** Comparison of Parent-Caregiver Perception Questionnaire overall and domain scores and caries status in primary dentitions.

Domains	Primary dentition	*n*	Mean score	SD	*t*	*p*
Oral symptoms	Caries free	62	3.44	3.37	−2.35	0.021*
	Caries present	35	5.14	3.57		
Functional limitation	Caries free	62	2.90	3.46	−2.27	0.025*
	Caries present	35	4.66	3.96		
Emotional well-being	Caries free	62	2.65	3.97	−0.37	0.715
	Caries present	35	2.94	3.60		
Social well-being	Caries free	61	1.05	2.50	−1.15	0.253
	Caries present	35	1.63	2.14		
P- CPQ score	Caries free	61	9.82	10.40	−2.13	0.035*
	Caries present	35	14.37	9.42		

P-CPQ, Parent-Caregiver Perception Questionnaire; SD, standard deviation.

* , Significant *p* value.

**TABLE 4:** Comparison of Parent-Caregiver Perception Questionnaire overall and domain scores and caries status in permanent dentitions.

Domains	Permanent dentition	*n*	Mean	SD	*t*	*p*
Oral symptoms	Caries free	30	3.17	2.63	−2.29	0.026*
	Caries present	38	5.05	3.87		
Functional limitation	Caries free	30	3.33	3.17	−1.39	0.169
	Caries present	38	4.55	3.89		
Emotional well-being	Caries free	30	3.27	2.95	−1.12	0.267
	Caries present	38	4.26	4.11		
Social well-being	Caries free	30	2.67	2.94	1.76	0.083
	Caries present	37	1.57	2.17		
P- CPQ score	Caries free	30	12.43	8.19	−1.26	0.212
	Caries present	37	15.22	9.57		

P-CPQ, Parent-Caregiver Perception Questionnaire; SD, standard deviation.

* , Significant *p* value.

Spearman Rho correlation coefficient was used to measure the extent of the association between dental caries and P-CPQ and global rating scores and the results are displayed in Table 5. The number of teeth affected by dental caries in the primary dentition was significantly correlated with OS, FL and SWB domains. However, the strength of the association was mild (ρ coefficient ranged between 0.23 and 0.25). Furthermore, the number of teeth affected by dental caries was also found to be moderately correlated with the global rating score and the overall oral health rating (ρ = 0.349; *p* = 0.005).

**TABLE 5:** Correlation between decayed, missing and filled teeth, global rating scores and total Parent-Caregiver Perception Questionnaire scores.

Variable	dmft		DMFT	
	ρ**	*p*	ρ**	*p*
Oral symptoms	0.246	0.034*	0.051	0.793
Functional limitation	0.231	0.041*	−0.091	0.511
Emotional well-being	0.078	0.494	0.054	0.692
Social well-being	0.252	0.038*	−0.24	0.103
**P-CPQ**	**0.154**	**0.273**	**-0.256**	**0.261**
Global rating-oral health	0.349	0.005*	0.349	0*
Global rating-overall well-being	0.02	0.84	0.117	0.339

** , Spearman’s correlation coefficient;

* , significant at *p* < 0.05.

dmft or DMFT, decayed, missing and filled teeth; P-CPQ, Parent-Caregiver Perception Questionnaire.

Bold text indicate the correlation between overall P-CPQ score and dental caries.

### Caregivers’ perceptions

When the caregivers were asked to comment on the effect of any oral condition on the overall well-being of the child, 60.7% (*n* = 91) caregivers reported that their children’s overall well-being was not affected by the oral conditions. The majority (56.7%) of the caregivers rated the children’s overall oral health status as average and only 12% rated it to be poor.

Spearman’s correlation coefficient was also used to assess construct validity of the study instrument (questionnaire). There was a strong correlation between the P-CPQ scores and the global rating of overall well-being scores (ρ = 0.56; *p* ≤ 0.001) and global rating of oral health score (ρ = 0.653; *p* < 0.001), respectively.

The impact of the socio-demographic factors of the caregiver, the caries status of the children and P-CPQ scores was evaluated using the linear regression model. The dependent variables were added into the equation in a stepwise form. The results displayed in Table 6 showed that there was no significant relationship between all the variables that were entered into the regression model and the P-CPQ scores.

**TABLE 6:** Linear regression of socio-demographic variables of the caregiver, decayed, missing and filled teeth of the children and Parent-Caregiver Perception Questionnaire.

Model	Variables	*B*	s.e.	*t*	*p*	Confidence interval	
						Low	Upper
-	(Constant)	−1.46	17.78	−0.08	0.94	−43.5	40.57
1	Caregiver age group	0.52	3.27	0.16	0.88	−7.21	8.26
2	Relationship	−3.99	4.17	−0.96	0.37	−13.85	5.87
3	Disability	1.1	2.13	0.52	0.62	−3.93	6.13
4	Level of education	8.4	4.47	1.88	0.10	−2.17	18.98
5	Employment	−5.48	4	−1.37	0.21	−14.94	3.98
6	dmft	−0.4	1.08	−0.37	0.72	−2.96	2.16
7	DMFT	4.81	3.50	1.38	0.21	−3.47	13.09

dmft or DMFT, decayed, missing and filled teeth; s.e., standard error.

## Discussion

Children with special needs are faced with the daily burden of dealing with the negative impact of their individual disabilities, and more specifically, the effect of these disabilities on oral health. Caregivers in this study were predominantly women and this is consistent with the findings of previous studies on caregivers’ perceptions of OHRQoL (Abanto et al. 2014; Baghdadi & Muhajarine 2014; Kumar, Kroon & Lalloo 2014). The reason for this has been attributed to the worldwide societal norm of women being regarded as primary caregivers (American Psychological Association 2015; Hlabyago & Ogunbanjo 2009). The gender distribution of the child participants favoured males rather than females. A possible reason for the male predominance in the CSNs could be that boys are more genetically predisposed to having one form or another of disability than are girls (Werling & Geschwind 2013).

### Caries status

Dental caries is a major public health problem in South Africa. Several reports have shown the prevalence of dental caries to be higher in children with disabilities compared to the general population (Nemutandani et al. 2013; Purohit, Acharya & Bhat 2010; Shyama 2001). Conversely, this current study and an earlier study by Nqcobo et al. (2012) found caries prevalence to be lower in CSNs in Johannesburg. Each child had at least one carious lesion. This is lower than the caries prevalence reported in the general population of South African children which is 60.3% in 6 year olds. Furthermore, this prevalence is lower than those reported by Shukla et al. (2014), in India (76%) and by Abanto et al. (2014), in Brazil (55%). The reason for the lower prevalence in this cohort studied could be that the special needs school had an established daily tooth brushing routine after lunch and the caregivers who attended the outreach sites were already exposed to ongoing oral health education at the outreach sites during support group discussions.

The highest caries prevalence was found in the primary dentition of the epilepsy group (83.30%) followed by the autism group (75%). Similarly, Gurbuz and Tan (2010) found the caries prevalence in children with epilepsy in Turkey to be high (96.7%). The reason for the high prevalence among this group may be as a result of medications which predispose them to gingival hyperplasia and dry mouth (Ghafoor, Rafeeq & Dubey 2014). This, in turn, facilitates plaque retention and ultimately increases the risk for dental caries (Ghafoor et al. 2014). The autism group also had a high caries prevalence in the current study, which is similar to the findings by Jaber et al. (2011). The reason for this high prevalence could be that children with autism have multiple medical and behavioural problems, which make their oral hygiene care extremely difficult (Lewis et al. 2015). It has been reported that children with autism prefer soft and sweetened foods. They also tend to pouch food inside the mouth instead of swallowing it because of poor tongue coordination, thus increasing the susceptibility to caries (Jaber 2011). In this study cohort, caution needs to be exercised when interpreting the high prevalence recorded for epilepsy and autistic groups because of the small sample sizes.

In keeping with other studies (Altun et al. 2010; Purohit et al. 2010), the prevalence of untreated caries among children with disabilities such as Down syndrome, cerebral palsy, epilepsy and other complex disabilities in this study was high. The reason may be because of lack of access to oral health services, long waiting lists for general anaesthesia and caregiver’s perception of oral health care as not being as much a priority as general health problems (Lewis et al. 2015).

### Oral-health-related quality of life

The present study showed that all the caregivers of CSNs reported that dental caries had a negative impact on OHRQoL outcomes as measured by P-CPQ scores. This is similar to the studies conducted by Abanto et al. (2012) and Pani et al. (2013) which showed a negative impact of oral diseases on OHRQoL of children with cerebral palsy and autism. All the caregivers, irrespective of the disabilities, reported that OS and FL domains in the primary dentition had an impact on the OHRQoL. This could be because of the consequences of untreated caries which may have progressed into the pulp and periapical tissues resulting in severe pain. The pain resulting from untreated dental caries has an impact on feeding and sleeping which may alert the caregiver to the extent of the pain.

### Caregivers’ perceptions

It is important to note that while the caregivers have reported that dental caries had a negative impact on the OHRQoL of CSN, overall well-being of the children was not significantly affected by oral conditions and the children’s overall oral health status was rated to be average. In contrast, Abanto et al. (2012) found that a lower proportion of caregivers rated their children’s oral health as excellent. The reason could be that the overall well-being of the children is mostly perceived by caregivers to be affected by the severity of the disability rather than the oral condition. This highlights the caregivers’ lack of awareness on the importance of oral health for general health. Oral health is not considered to be life-threatening by most population groups. It is also not considered important to overall well-being, especially when oral health diseases co-exist with some debilitating illnesses or disabilities. It is important that policy makers and health educators begin to make concerted efforts to educate the growing population of caregivers on the importance of oral health and the role it plays in general health. Pani et al. (2013) also support this assertion.

## Conclusion and recommendations

The caregivers did not perceive oral conditions to affect the overall well-being of the children and this could have been attributed to a lack of awareness of the importance of the contribution oral health to general health. There was a high burden of untreated dental caries and it is thus recommended that caregiver and teacher education on oral health should be expanded in all special needs schools and primary health care centres, especially the maternal and child health centres. This would facilitate early detection of oral diseases and early intervention. Oral health education and promotion, especially designed for individual disabilities, and proven preventive methods such as the use of fissure sealants, are recommend to be implemented in this group of children.
